# Feasibility of detecting cognitive impairment using Natural Language Processing‐AI analysis of digital voice recordings of a brief neuropsychological battery obtained by a smartphone

**DOI:** 10.1002/alz70861_108806

**Published:** 2025-12-23

**Authors:** Sam A. Murdock, Neguine Rezaii, Alexa Goldstein, Erik Larsen, Olivia Murton, Brad C. Dickerson, Bonnie Wong

**Affiliations:** ^1^ Frontotemporal Disorders Unit, Massachusetts General Hospital, Boston, MA USA; ^2^ Frontotemporal Disorders Unit and Massachusetts Alzheimer’s Disease Research Center, Department of Neurology, Massachusetts General Hospital and Harvard Medical School, Boston, MA USA; ^3^ Sonde Health, Boston, MA USA; ^4^ Department of Psychiatry, Massachusetts General Hospital, Boston, MA USA

## Abstract

**Background:**

Mobile‐based cognitive assessments offer a promising low‐burden approach to screen for cognitive impairment, but their tolerability and diagnostic accuracy remain underexplored (Vanderlip et al., 2024). This study evaluated the feasibility of using a mobile app to obtain voice‐based neuropsychological testing and detect cognitive impairment through an NLP‐AI analysis of memory performance.

**Methods:**

34 participants with MCI or mild dementia (mean age = 66.6 ± 8.0 years, 44% female; CDR £ 1) and 20 cognitively healthy controls (mean age = 68.8 ± 4.9 years, 60% female) completed a brief battery of verbal neuropsychological tests (assessing executive functioning, memory, and language) using the Sonde One mobile app. Responses were captured via at‐home voice recordings. Neuropsychological test scores were summed to generate a composite score for each participant who recorded at least five of seven tasks. Using a large language model‐based algorithm, we measured verbal memory performance by calculating the language informative index (LII; Bayat et al., 2024), which assessed the similarity between the original narrative and immediate and delayed recalls of the story. ROC curve analysis was conducted to assess the accuracy of the composite score and LII in distinguishing patients from controls.

**Results:**

27 patients (79%) and 19 controls (95%) successfully completed at least five neuropsychological tasks either independently or with caregiver support. ROC curve analyses demonstrated strong diagnostic accuracy of the neuropsychological composite score in distinguishing patients from controls (AUC = 0.99, 95% CI = 0.96‐1.00). Diagnostic classification accuracy using story recall LII was also strong (Immediate Recall: AUC = 0.96, 95% CI = 0.91‐1.00; Delayed Recall: AUC = 0.93, 95% CI = 0.86‐1.00).

**Conclusions:**

Our findings indicate that remote, at‐home cognitive assessment via mobile app is a feasible method for detecting MCI and mild dementia. The strong performance of LII‐based memory analysis suggests it may serve as an effective, low‐burden tool for streamlining cognitive assessments in both remote and clinical settings. Additional analyses are underway to determine whether LII can be used to discriminate among dementia syndromes and severity levels.

